# What Could Be Responsible for Some Mosquito-Borne Diseases? Is It Poverty, Gender Inequality, Underdevelopment, Globalization, or Climate Change? Which One(s)?

**DOI:** 10.1155/jotm/5405719

**Published:** 2025-09-30

**Authors:** Elif Nur Yildirim-Ozturk

**Affiliations:** ^1^Konya Provincial Health Directorate, Selcuklu, Konya, Turkey; ^2^Necmettin Erbakan University, Meram, Konya, Turkey

**Keywords:** climate change, DALY, gender inequality, mosquito-borne disease, poverty

## Abstract

**Background:**

Mosquito-borne diseases are a major cause of mortality and disease burden worldwide. This study aimed to assess the trends in total disability-adjusted life years (DALYs) due to five mosquito-borne diseases, as well as their association with poverty, gender inequality, underdevelopment, globalization, and climate change, both globally and for the period from 1990 to 2021.

**Methods:**

This ecological time-series study with a longitudinal analytical framework used a total of 27 variables obtained from different sources. The dependent variable of the study was mosquito-borne DALYs. The trend of the numerical variables over time was analyzed using joinpoint regression. The relationships between the dependent variable and the independent variables were examined using univariate linear regression, LASSO regression, and ridge regression. *p* < 0.05 was considered statistically significant.

**Results:**

During the study period, mosquito-borne DALYs decreased by 1.13 per 100,000 persons per year. The LASSO regression model explained 97.9% of the variability in mosquito-borne DALYs. Poverty headcount ratio at $6.85 a day, share of seats in parliament (female), global greenhouse gas emission, and Gender Inequality Index were found to be the most influential variables on mosquito-borne DALYs, respectively. When the optimum lambda, *R*^2^, MSE, and RMSE values were analyzed, the LASSO regression model was found to be more compatible than ridge regression for this data set.

**Conclusion:**

The results demonstrate that mosquito-borne DALYs are primarily driven by poverty but are also influenced by gender inequality and climate change. These results highlight the urgent need for integrated and multifaceted public health strategies that go beyond traditional vector control methods.

**Summary**

• This study analyzes the global trend of DALYs caused by the five leading mosquito-borne diseases over the 32-year period from 1990 to 2021.
• It also investigates the influence of variables related to poverty, gender inequality, underdevelopment, globalization, and climate change using two regression models.
• Poverty was found to be the most influential factor affecting mosquito-borne DALYs, followed by gender inequality and climate change.
• Prioritizing these determinants in both current and future public health policies and programs would offer significant global benefits.

## 1. Introduction

Mosquito-borne diseases are caused by pathogens transmitted by mosquitoes, which are arthropod vectors. The mosquito, a blood-sucking flying insect, is found worldwide. Because mosquitoes are present year-round in tropical and subtropical regions, but only seasonally in other regions, mosquito-borne diseases pose a significant public health risk worldwide [1]. Mosquito-borne diseases include parasitic diseases, such as malaria and lymphatic filariasis, and viral infections, such as dengue, Zika virus disease, yellow fever, chikungunya, Japanese encephalitis, and West Nile fever [2].

Vector-borne diseases account for 17% of all infectious diseases and cause more than 700,000 deaths annually [2]. According to the Global Vector Control Response 2017–2030 report, there were more than 347 million cases and 447,000 deaths from mosquito-borne diseases in 2016 [3]. In 2023, there were 263 million cases and 597,000 deaths due to malaria, one of the diseases included in this study. The incidence of the disease was 60.4 cases per 1000 population at risk, and the mortality rate was 13.7 per 100,000. [4]. Yellow fever, dengue, Zika, lymphatic filariasis, chikungunya, Japanese encephalitis, and West Nile fever also pose a serious global health burden. Each has distinct risks, such as disability, outbreaks, or severe outcomes [5–11]. In addition, malaria, yellow fever, Japanese encephalitis, and West Nile fever can cause illness and death in some animal species, which may be a warning sign [4, 5, 10, 11].

Mosquito-borne diseases, which can have a significant impact on both the individual and society, are influenced by a variety of factors that play a critical role in their breeding and transmission dynamics and epidemiology, as evidenced in the literature. These factors can be summarized as climate and environmental factors [12], mosquito–pathogen–host interactions [13, 14], urbanization [15], human behavior [16], travel and migration [17, 18], vector control and public health infrastructure [19, 20], and socioeconomic status [21].

A review of the literature shows that the factors potentially associated with mosquito-borne diseases have mostly been studied separately. The effect of mosquito-borne diseases on populations is widespread, and it can be assessed in the changes of disability-adjusted life years (DALYs). DALYs account for both premature mortality and years lived with disability, providing a more comprehensive view of disease burden than incidence or prevalence alone. This is particularly relevant for mosquito-borne diseases, which cause both deaths and long-term health consequences.

The study aimed to assess trends in total DALYs due to mosquito-borne diseases and their association with poverty, gender inequality, underdevelopment, globalization, and climate change globally, over the period 1990–2021.

## 2. Materials and Methods

### 2.1. Study Type and Permissions

This was an ecological time-series study with a longitudinal analytical framework, as it used aggregate-level data analyzed over a 32-year period. The research was carried out between 01.03.2024 and 01.11.2024. The research was conducted completely through publicly available sources, and there was no direct human contact. Therefore, no ethical or institutional approval was required. In addition, appropriate references for all variables used in the study are cited in the article.

### 2.2. Study Settings

The main question of the research is which of the following factors has the greatest impact on mosquito-borne diseases: poverty, inequality, development, globalization, or climate? To answer this question, variables that may be related to these five different independent variables were selected. Both the dependent and independent variables were selected from reliable data sources. The data used in the research were collected for 32 years from 1990 to 2021 and recorded annually in a data collection form. The study examined a total of 27 variables, which are as follows: total DALYs attributable to malaria, yellow fever, dengue, Zika, and lymphatic filariasis (mosquito-borne DALYs), Konjunkturforschungsstelle (KOF) Globalization Index, the poverty headcount ratio at $2.15/3.65/6.85 a day (% of population), poverty gap at $2.15/3.65/6.85 a day, annual anomalies in global land and ocean surface temperature, annual anomalies in global land surface temperature, annual anomalies in global ocean surface temperature, global emission of CO_2_, global greenhouse gas emission, Human Development Index (HDI) and its components, and Gender Inequality Index (GII) and its components.

### 2.3. Dependent and Independent Variables

The sum of DALYs attributable to malaria, yellow fever, dengue, Zika, and lymphatic filariasis, standardized for age per 100,000 population, was the study's dependent variable. This study refers to this variable as “mosquito-borne DALYs.” DALYs for other mosquito-borne diseases, such as chikungunya, Japanese encephalitis, and West Nile fever, were unavailable from the source, so these diseases could not be included. A DALY represents the loss of 1 year of healthy life, combining both losses due to premature death and losses in quality of life due to illness or disability. DALY is a quantitative measure of disease burden [22]. The DALYs used in the study were taken from the Global Burden of Disease Study (GBD) 2021. The GBD is the largest and most up-to-date pool of epidemiological data provided by the Institute for Health Metrics and Evaluation at the University of Washington. The DALYs in this dataset are based on a variety of sources, including primary sources, independent research studies, government reports, vital statistics records, verbal autopsies, disease registries, health projects, and census data [23, 24].

#### 2.3.1. Variable Definitions

KOF Globalization Index (KOF_GI) is an index that measures the level of globalization of countries around the world. It focuses on three main dimensions: economic, social, and political globalization. This index is used to assess the international integration and interdependence of countries [25–27].

The poverty headcount ratio at $2.15/3.65/6.85 a day (%) is a measure of poverty defined by the World Bank. This expresses the percentage of the population living below the defined thresholds ($2.15/3.65/6.85). $2.15/3.65/6.85 are the thresholds for extreme, moderate, and high-moderate poverty, respectively. These thresholds are used to measure poverty in low-, lower-middle-, and upper-middle-income countries, respectively [28].

Poverty gap at $2.15/3.65/6.85 a day (%) shows the mean amount of income that the population living below the poverty threshold needs per day to reach the poverty threshold and how much their income is below the threshold [29].

Annual anomalies in global land surface temperature and annual anomalies in global ocean surface temperature are calculated for land and ocean to monitor climate change and temperature trends over time. These values express how much the global mean temperature values in a year deviate from the long-term mean in degrees Celsius [30].

Global emission of CO_2_ is a measure of the amount of carbon dioxide (CO_2_) emitted into the atmosphere from various sources around the world, expressed in billion tons. As a major greenhouse gas, CO_2_ plays a key role in global warming and climate change [31, 32].

Global greenhouse gas emission is an indicator of the amount of greenhouse gases emitted into the atmosphere from all sources worldwide, expressed in billion tons of CO_2_ equivalent. The main greenhouse gases that trap heat in the atmosphere are CO_2_, methane (CH_4_), nitrous oxide (N_2_O), and fluorinated gases [32, 33].

HDI is an index developed by the United Nations Development Programme (UNDP) that measures a country's level of human development. The index is calculated using Gross National Income (GNI) per capita, expected years of schooling, average years of schooling, and life expectancy at birth and can range from 0 to 1. As the index values approach 1, human development increases [34].

GII is an index developed by UNDP to measure gender inequality. The GII assesses a country's level of gender inequality by comparing the achievements and opportunities of women and men in several key areas. This shows the potential loss of human development due to the disparity between women's and men's outcomes in three dimensions: reproductive health, empowerment, and economic status. The reproductive health dimension in the GII takes into account maternal mortality and adolescent fertility rates; the empowerment dimension takes into account the number of women and men in parliament and the level of secondary education of adults aged 25 and over; and the economic status dimension takes into account the labor force participation rates of women and men aged 15 and over. It can range from 0 to 1, and inequality increases as the index values approach 1 [35].

### 2.4. Statistical Analysis

Data analyses were conducted using SPSS Inc. PASW Statistics for Windows, Version 18.0. (Chicago (IL): SPSS Inc.; 2009), the Joinpoint Regression Program (Version 4.9.1.0; National Cancer Institute), and RStudio (Version 2023.09.1 + 494; Posit Software, PBC). The trend of numerical variables over time was analyzed using joinpoint regression, with the joinpoint interval set as a minimum of 2 and the number of joinpoints set as a maximum of 6. The joinpoints were selected by the researcher, taking into account the direction of the trend and statistical significance, and aiming for the minimum number of joinpoints. The standard error values required for analysis were calculated as the mean of the standard error for each variable in the database, containing 32 years of data. The fit of the dependent variable–independent variable relationship to the linear model was tested by curve estimation. High correlation and multicollinearity between numerical variables were predicted, and therefore, multiple linear regression was avoided. The relationships between the dependent and independent variables were examined using Least Absolute Shrinkage and Selection Operator (LASSO) regression and ridge regression. The independent variables included in LASSO and ridge regression models were selected based on their *R*^2^ values from univariate linear regression analyses. To ensure comprehensive representation of the key domains, at least one variable was deliberately chosen from each of the following categories: poverty, inequality, development, globalization, and climate. For the categories of development and inequality, both the main index (HDI and GII, respectively) and the component variable with the highest *R*^2^ value within each category were included in the models. To avoid overfitting the model and to determine the most optimal value of the regularization coefficient (*λ*), 10-fold cross-validation was applied. Variables to be included in LASSO and ridge regression were subjected to z-standardization. The LASSO and ridge regression models were compared, and the model offering better, more explanatory results was preferred. Next, a sensitivity analysis was performed to test the stability of the chosen model under varying conditions. *p* < 0.05 was considered statistically significant.

## 3. Results

**Figure 1:**
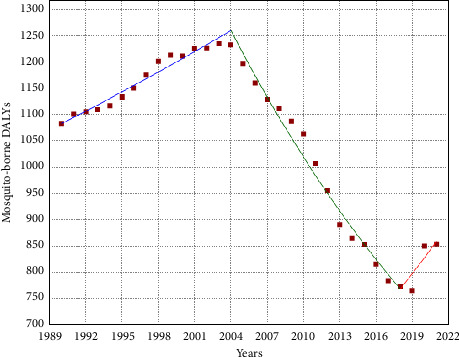
Trend of mosquito-borne DALYs from 1990 to 2021.

The variables used in the study and the means of the variables are shown in Table 1. Trends in the variables included in the study over the 32-year period from 1990 to 2021 were analyzed and presented in Supporting Table 1. For mosquito-borne DALYs, a statistically significant decrease of 1.13 per 100,000 persons per year was observed (*p* < 0.05). As shown in Figure 1, a total of three statistically significant trends and two joinpoints were identified. Mosquito-borne DALYs first showed an increasing trend, then a decreasing trend, and finally an increasing trend again. None of the other variables showed similar trends in similar years for mosquito-borne DALYs.

The details of the univariate linear regression models obtained when the dependent variable was mosquito-borne DALYs and the other 26 variables were included as independent variables are shown in Supporting Table 2. As a result of the analysis, the variable that had the greatest impact on mosquito-borne DALYs was the poverty headcount ratio at $6.85 a day, with an adjusted *R*^2^ of 0.821. Additionally, other variables that represent climate change, human development, inequality, and globalization associated with mosquito-borne DALYs were identified: annual anomalies in global land and ocean surface temperature, global greenhouse gas emission, HDI and GNI per capita, GII, share of seats in parliament (female) and share of seats in parliament (male), and KOF_GI.

LASSO and ridge regression models were constructed with nine independent variables to explain the dependent variable of mosquito-borne DALYs. The optimal lambda values and analysis results of the models are shown in Table 2.

According to the LASSO regression model, the optimal lambda value was 0.001, *R*^2^ 0.979, MSE 0.020, and RMSE 0.142. The model explained 97.9% of the variability in mosquito-borne DALYs. The poverty headcount ratio at $6.85 a day, share of seats in parliament (female), global greenhouse gas emission, and GII were found to be the most influential variables on mosquito-borne DALYs. The coefficients of the variables HDI, GNI per capita, share of seats in parliament (male), and KOF_GI were 0.

According to the ridge regression model, the optimal lambda value was 0.089, *R*^2^ 0.884, MSE 0.112, and RMSE 0.335. The model explained 88.4% of the variability in mosquito-borne DALYs. The poverty headcount ratio at $6.85 a day, KOF_GI, share of seats in parliament (male), and share of seats in parliament (female) were found to be the most influential variables on mosquito-borne DALYs.

When examining the optimal lambda, *R*^2^, adjusted *R*^2^, MSE, and RMSE values, the LASSO regression model was found to be more compatible for this dataset. Sensitivity analysis with 100 replicates was performed for the LASSO regression model using 80% of the dataset. In each replicate, 10-fold cross-validation was applied, and the variables with nonzero coefficients corresponding to the optimal lambda value were recorded in the model. Four of the five variables with nonzero coefficients in the LASSO model in Table 2 (between 76% and 100%) were highly reselected in the sensitivity analysis and showed stability. KOF_GI that was included in the original model but not selected was selected 82% of the time and considered a stable variable that could contribute to the model. In contrast, the global greenhouse gas emission was selected only 43% of the time and its contribution to the model was limited and sensitive to sampling. The mean lambda values used in the sensitivity analysis were 0.00229 ± 0.00147, varying within a narrow range (0.00008–0.00589).

## 4. Discussion

In this study, the poverty headcount ratio at $6.85 a day was identified as the variable most associated with mosquito-borne DALYs. The high coefficient of this variable in univariate linear regression and in both LASSO and ridge regressions is noteworthy. This variable is followed by the poverty headcount ratio at $3.65 a day. Although these two results suggest a relationship between the dependent variable and income status, it should be noted that the coefficient for GNI per capita, which is also an income-related variable and reflects national average income rather than the extent of poverty, is zero in the LASSO regression. This indicates that mosquito-borne DALYs are more strongly associated with poverty than with general income level. In light of these findings, it can be said that mosquito-borne DALYs are mainly influenced by poverty. Limited access to healthcare, poor infrastructure conditions, and limited resources for vector and disease control may be the reasons for this situation. Some studies in the literature support the relationship between poverty and mosquito-borne diseases [36–38]. The current study also supports this association. However, a systematic review published in 2015 failed to show a clear relationship between dengue and poverty, highlighting the need for more research in this area [39].

The second variable affecting mosquito-borne DALYs was share of seats in parliament (female), and the fourth was GII. However, it is important to note that the variable share of seats in parliament (male), which is excluded from the LASSO regression model, is closely related to the variables share of seats in parliament (female) and GII. This is because, as share of seats in parliament (female) increases, share of seats in parliament (male) decreases. In other words, although share of seats in parliament (male) is a variable outside the model, it is still closely related to the important variables in the model. This study shows that increasing female representation in parliament and decreasing gender inequality appear to increase the disease burden. The reasons for this should be interpreted with caution. Low representation of women in parliament and high gender inequality may actually indicate the depth of inequalities. This may lead to a lack of access to health services and underreporting of existing diseases. The positive impact of increased female representation in parliament and reduced gender inequality may have contributed to a higher recorded disease burden by improving diagnosis and case registration. These positive effects may include improved data collection and reporting, policy changes, increased social awareness and sensitivity, and improved access to health services. Gender inequality is known to affect the health of both women and men [40]. Two studies have examined the relationship between GIIs and life expectancy at birth, healthy life expectancy at birth, and noncommunicable disease mortality [41, 42]. One study reports on the negative impact of past epidemics, including the 2016 Zika virus outbreak, on gender equality [43]. In addition, two studies evaluating the control measures and campaigns implemented during the Zika virus outbreak and the role of women in dengue prevention reported that gender inequality was exacerbated and gender roles were reinforced [44, 45]. Additionally, increased female representation in parliaments is associated with better health outcomes, including lower infant mortality rates and higher vaccination rates [46, 47].

Global greenhouse gas emission was identified as the third variable influencing mosquito-borne DALYs. Annual anomalies in global land and ocean surface temperature ranked behind global greenhouse gas emissions in both univariate and multivariate analyses. Thus, mosquito-borne DALYs appear to be more influenced by atmospheric changes than by land or ocean temperature anomalies. The rise in temperature caused by increasing greenhouse gases in the atmosphere may support and expand the habitat of mosquitoes. Increasing greenhouse gases in the atmosphere can cause changes in rainfall patterns, creating areas suitable for mosquito breeding [48]. Air pollution caused by increasing greenhouse gases in the atmosphere can affect human migration patterns and increase the burden on health services [49, 50]. All of these mechanisms may play a role in the relationship between mosquito-borne DALYs–global greenhouse gas emissions. This research suggests that the first climate change goal in attempting to reduce mosquito-borne DALYs would be to reduce global greenhouse gas emissions. There are also studies linking mosquito-borne diseases and climate change [51, 52]. However, it is emphasized that there are different topics to be investigated in this area [53, 54]. Furthermore, compliance with the Paris Agreement, to which most countries are parties, and the implementation of national commitments may be expected to reduce greenhouse gas emissions and mosquito-borne DALYs.

In this study, in the LASSO regression model, in the presence of other variables contributing to the model, the coefficient of the variables HDI, GNI per capita, share of seats in parliament (male), and KOF_GI were calculated as zero. It can therefore be concluded that these four variables do not have a direct and significant effect on mosquito-borne DALYs. However, these variables may have been excluded from the model because they are highly correlated with the variables poverty headcount ratio at $6.85 a day, share of seats in parliament (female), global greenhouse gas emission, and GII, which are the most highly correlated with the dependent variable. In addition, although four of the five variables in the model showed stability during the sensitivity analysis, one of them (global greenhouse gas emission) was sensitive to sampling and its contribution to the model was limited. At this point, it was determined that the KOF_GI variable, which was not included in the LASSO regression model, could make a stable contribution to the model. The researcher could not find any literature directly evaluating the relationship between globalization, KOF_GI, and mosquito-borne disease. In this respect, the relationship between globalization and mosquito-borne diseases can be considered open-minded. In the case of a plan/policy on mosquito-borne diseases, interventions related to HDI, GNI per capita, share of seats in parliament (male), and KOF_GI variables should not be completely ignored but should be considered as secondary rather than primary. In both the LASSO and ridge regressions, the intercept was near zero, with a small negative value. The reason for this situation may be that the data used in the model contain extreme values that cannot be changed/excluded because they are real data. Another possible reason is the presence of variables that have a negative value and are determined to have a strong impact on mosquito-borne DALYs. Additionally, the sign of some coefficients observed in the univariate regression models reversed in the regularized regression models. This may be attributed to differences in the underlying principles of univariate and regularized models, as well as to multicollinearity among variables. Regularization techniques reduce or set to zero the coefficients to increase precision, which can cause some variables to change direction.

### 4.1. Advantages and Limitations of the Study

This study examined trends in DALYs for five mosquito-borne diseases and the factors that most influence, or are likely to influence, these DALYs over a 32-year period worldwide. The factors considered are broad, ranging from poverty to gender inequality, human development, globalization, and climate change. The research was conducted with up-to-date data from reliable sources. The research used joinpoint regression, univariate linear regression, LASSO regression, and ridge regression techniques. These are the superior aspects of this study.

This study was based on population-level data rather than individual-level observations. Study data were obtained from several sources. Multicollinearity was expected among the independent variables studied. When evaluating and interpreting the results of the study, it should be taken into account that each variable may be related to the other. Since this study focused on the big picture, it may have overlooked the unique characteristics of each disease-mediating vector, such as ecological behavior and flight range. These are the limitations of the study.

## 5. Conclusion

As a result of this study, it was found that the dependent variable, mosquito-borne DALYs, first showed an increasing trend, then a decreasing trend, and then an increasing trend again. Using univariate linear regression, it was found that all of the independent variables explained approximately one-third of the variance in mosquito-borne DALYs (*R*^2^ = 0.296) and the variable with the highest explanatory rate was poverty headcount ratio at $6.85 a day. The most influential variables on mosquito-borne DALYs are poverty headcount ratio at $6.85 a day, share of seats in parliament (female), global greenhouse gas emission, and GII in LASSO regression and poverty headcount ratio at $6.85 a day, KOF_GI, share of seats in parliament (male), and share of seats in parliament (female) in ridge regression. For the data set used in the study, the LASSO regression model was found to be more valid than the ridge regression.

In public health programs implemented to reduce mosquito-borne diseases, issues such as vector control, vaccine development and use, public education and awareness, early diagnosis and rapid treatment of the disease, and global cooperation are commonly addressed and should continue to be prioritized. However, an initiative to control/reduce mosquito-borne DALYs should prioritize poverty reduction, increasing women's representation in leadership, reducing greenhouse gas emissions, and reducing gender inequality.

## Figures and Tables

**Table 1:** Means of the variables used in the study.

	Mean ± std dev	Years	Period of data
Mosquito-borne DALYs	1052.70 ± 157.42	1990–2021	Annually
Poverty headcount ratio at $2.15 a day	21.92 ± 9.99	1990–2021	Annually
Poverty headcount ratio at $3.65 a day	41.74 ± 11.69	1990–2021	Annually
Poverty headcount ratio at $6.85 a day	61.09 ± 8.77	1990–2021	Annually
Poverty gap at $2.15 a day	7.14 ± 3.78	1990–2021	Annually
Poverty gap at $3.65 a day	17.65 ± 6.87	1990–2021	Annually
Poverty gap at $6.85 a day	34.15 ± 8.42	1990–2021	Annually
Annual anomalies in global land surface temperature	0.95 ± 0.39	1990–2021	Annually
Annual anomalies in global ocean surface temperature	0.48 ± 0.15	1990–2021	Annually
Annual anomalies in global land and ocean surface temperature	0.63 ± 0.22	1990–2021	Annually
Global emission of CO_2_	29.71 ± 5.27	1990–2021	Annually
Global greenhouse gas emission	42.58 ± 7.08	1990–2021	Annually
HDI	0.67 ± 0.05	1990–2021	Annually
Life expectancy	69.22 ± 2.54	1990–2021	Annually
Expected years of schooling	11.05 ± 1.29	1990–2021	Annually
Mean years of schooling	7.42 ± 0.85	1990–2021	Annually
Gross National Income per capita	12687.25 ± 2453.45	1990–2021	Annually
GII	0.53 ± 0.04	1990–2021	Annually
Maternal mortality ratio	285.79 ± 62.45	1990–2021	Annually
Adolescent birth rate	57.81 ± 10.74	1990–2021	Annually
Share of seats in parliament (female)	17.13 ± 4.89	1990–2021	Annually
Share of seats in parliament (male)	82.87 ± 4.89	1990–2021	Annually
Population with at least some secondary education (25 years and older) (female)	50.68 ± 8.61	1990–2021	Annually
Population with at least some secondary education (25 years and older) (male)	59.84 ± 7.95	1990–2021	Annually
Labor force participation rate (15 years and older) (female)	47.48 ± 1.40	1990–2021	Annually
Labor force participation rate (15 years and older) (male)	76.17 ± 1.74	1990–2021	Annually
KOF_GI	54.19 ± 6.36	1990–2021	Annually

**Table 2:** The results of LASSO and ridge regression models constructed to explain the variable mosquito-borne DALYs.

Variables and model parameters	LASSO	Ridge
Lambda	0.001	0.089
Intercept	−0.000000002929518	−0.0000001210993
Poverty headcount ratio at $6.85 a day	3.110	0.668
Annual anomalies in global land and ocean surface temperature	0.117	0.013
Global greenhouse gas emission	0.543	−0.146
HDI	0	0.043
Gross National Income per capita	0	−0.180
GII	−0.454	−0.082
Share of seats in parliament (female)	1.140	−0.205
Share of seats in parliament (male)	0	0.206
KOF_GI	0	0.458
*R* ^2^	0.979	0.884
Adjusted *R*^2^	0.975	0.847
MSE	0.020	0.112
RMSE	0.142	0.335

## Data Availability

The data that support the findings of this study are available in the Institute for Health Metrics and Evaluation, GBD results at https://vizhub.healthdata.org/gbd-results/, References 23, 25–35 from the main document file. These data were derived from the following resource available in the public domain: Institute for Health Metrics and Evaluation, GBD results, https://vizhub.healthdata.org/gbd-results/.

## Supporting Information

Supporting Table 1: Trends of the variables from 1990 to 2021. Supporting Table 2: Univariate linear regression models for the dependent variable mosquito-borne DALYs.
