# Supplementary material for: What Could Be Responsible for Some Mosquito-Borne Diseases? Is It Poverty, Gender Inequality, Underdevelopment, Globalization, or Climate Change? Which One(s)?
Source: J Trop Med. 2025 Sep 30;2025:5405719. doi: 10.1155/jotm/5405719 (PMC12549193; doi:10.1155/jotm/5405719)
Supplement: Supporting Information — Additional supporting information can be found online in the Supporting Information section. [file 5405719.f1.docx]

**Supporting Table 1.** Trends of the variables from 1990 to 2021

|  | Trend 0 | | Trend 1 | | Trend 2 | | Trend 3 | | Trend 4 | |
| --- | --- | --- | --- | --- | --- | --- | --- | --- | --- | --- |
|  | Years | APC | Years | APC | Years | APC | Years | APC | Years | APC |
| Mosquito borne DALYs | 1990-2021 | -1,13* | 1990-2004 | 1,09* | 2004-2018 | -3.47* | 2018-2021 | 3.82* |  |  |
| Poverty headcount ratio at $2.15 a day | 1990-2021 | -4,47* | 1990-2002 | -2.73* | 2002-2018 | -6.86* | 2018-2021 | 3.32 |  |  |
| Poverty headcount ratio at $3.65 a day | 1990-2021 | -2.76* | 1990-2002 | -1.11* | 2002-2019 | -4.17* | 2019-2021 | -0.85 |  |  |
| Poverty headcount ratio at $6.85 a day | 1990-2021 | -1,42* | 1990-2002 | -0.18* | 2002-2019 | -2.24* | 2019-2021 | -0.19 |  |  |
| Poverty gap at $2.15 a day | 1990-2021 | -5.29* | 1990-2002 | -3.79* | 2002-2014 | -8.35* | 2014-2021 | -0.91 |  |  |
| Poverty gap at $3.65 a day | 1990-2021 | -3.87* | 1990-2002 | -2.29* | 2002-2018 | -5.72* | 2018-2021 | 1.16 |  |  |
| Poverty gap at $6.85 a day | 1990-2021 | -2.47* | 1990-2001 | -0.94* | 2001-2019 | -3.49* | 2019-2021 | 0.28 |  |  |
| Annual anomalies in global land surface temperature | 1990-2021 | 3.51* |  |  |  |  |  |  |  |  |
| Annual anomalies in global ocean surface temperature | 1990-2021 | 2.72* |  |  |  |  |  |  |  |  |
| Annual anomalies in global land and ocean surface temperature | 1990-2021 | 3.17* |  |  |  |  |  |  |  |  |
| Global emission of CO_2_ | 1990-2021 | 1.86* | 1990-1999 | 1.10 | 1999-2012 | 2.75* | 2012-2021 | 0.41 |  |  |
| Global greenhouse gas emission | 1990-2021 | 1.76* | 1990-1999 | 1.04* | 1999-2012 | 2.46* | 2012-2021 | 0.69* |  |  |
| HDI | 1990-2021 | 0.73* | 1990-1993 | 0.47* | 1993-2014 | 0.80* | 2014-2019 | 0.51* | 2019-2021 | -0.34* |
| Life expectancy | 1990-2021 | 0.39* | 1990-2019 | 0.42* | 2019-2021 | -1.30* |  |  |  |  |
| Expected years of schooling | 1990-2021 | 1.24* | 1990-1997 | 0.78* | 1997-2014 | 1.49* | 2014-2021 | 0.48* |  |  |
| Mean years of schooling | 1990-2021 | 1.21* | 1990-1999 | 1.82* | 2000-2016 | 1.11* | 2016-2021 | 0.86* |  |  |
| Gross National Income per capita | 1990-2021 | 2.08* | 1990-1994 | 0.10 | 1994-2018 | 2.25* | 2018-2021 | 0.37 |  |  |
| GII | 1990-2021 | -0.87* | 1990-1996 | -0.04 | 1996-2007 | -1.10* | 2007-2021 | -0.83* |  |  |
| Maternal mortality ratio | 1990-2021 | -2.26* | 1990-1992 | 1.85* | 1992-1997 | -0.83* | 1997-2015 | -2.90* | 2015-2021 | -0.23 |
| Adolescent birth rate | 1990-2021 | -1.94* | 1990-2001 | -1.49* | 2001-2005 | -3.78* | 2005-2021 | -1.51* |  |  |
| Share of seats in parlimant (female) | 1990-2021 | 3.08* | 1990-1996 | 0.01 | 1996-2008 | 3.90* | 2008-2021 | 2.83* |  |  |
| Share of seats in parlimant (male) | 1990-2021 | -0.61* | 1990-1997 | -0.03 | 1997-2011 | -0.68* | 2011-2021 | -0.82* |  |  |
| Population with at least some secondary education (25 years and older) (female) | 1990-2021 | 1.81* | 1990-2000 | 2.43* | 2000-2015 | 1.71* | 2015-2021 | 1.42* |  |  |
| Population with at least some secondary education (25 years and older) (male) | 1990-2021 | 1.39* | 1990-1999 | 2.37* | 1999-2015 | 1.27* | 2015-2021 | 0.83* |  |  |
| Labor force participation rate (15 years and older) (female) | 1990-2021 | -0.11 | 1990-1993 | -3.19* | 1993-2000 | 1.21* | 2000-2021 | -0.35* |  |  |
| Labor force participation rate (15 years and older) (male) | 1990-2021 | -0.24* | 1990-2005 | -0.27* | 2005-2013 | 0.11 | 2013-2021 | -0.37* |  |  |
| KOF_GI | 1990-2021 | 1,20* | 1990-2009 | 1,72* | 2009-2021 | 0,37* |  |  |  |  |

APC: Annually percentage change

* Statistically significance

**Supporting Table 2.** Univariate linear regression models for the dependent variable mosquito-borne DALYs

|  | Constant | Unstandardized B | Standardized Beta | Statistics for Standardized Beta | | %95 CI for Standardized Beta | | Model | | R^2^ | Adjusted R^2^ | Standard Error of the Estimate |
| --- | --- | --- | --- | --- | --- | --- | --- | --- | --- | --- | --- | --- |
|  |  |  |  | t | p | Lower | Upper | F | p |  |  |  |
| **Poverty related variables** | | | | | | | | | | | | |
| Poverty headcount ratio at $2.15 a day | 784.120 | 12.253 | 0.778 | 6.776 | <0.001 | 8.560 | 15.946 | 45.921 | <0.001 | 0.605 | 0.592 | 100.592 |
| Poverty headcount ratio at $3.65 a day | 571.776 | 11.521 | 0.855 | 9.049 | <0.001 | 8.921 | 14.121 | 81.876 | <0.001 | 0.732 | 0.723 | 82.866 |
| Poverty headcount ratio at $6.85 a day | 56.072 | 16.314 | 0.909 | 11.952 | <0.001 | 13.526 | 19.101 | 142.851 | <0.001 | 0.826 | 0.821 | 66.667 |
| Poverty gap at $2.15 a day | 841.367 | 29.608 | 0.711 | 5.544 | <0.001 | 18.702 | 40.515 | 30.740 | <0.001 | 0.506 | 0.490 | 112.462 |
| Poverty gap at $3.65 a day | 732.071 | 18.169 | 0.793 | 7.123 | <0.001 | 12.959 | 23.379 | 50.732 | <0.001 | 0.628 | 0.616 | 97.548 |
| Poverty gap at $6.85 a day | 510.243 | 15.883 | 0.849 | 8.807 | <0.001 | 12.200 | 19.566 | 77.568 | <0.001 | 0.721 | 0.712 | 84.509 |
| **Climate change related variables - Land and ocean** | | | | | | | | | | | | |
| Annual anomalies in global land surface temperature | 1328.636 | -289.604 | -0.712 | -5.561 | <0.001 | -395.968 | -183.241 | 30.921 | <0.001 | 0.508 | 0.491 | 112.295 |
| Annual anomalies in global ocean surface temperature | 1435.376 | -791.578 | -0.736 | -5.962 | <0.001 | -1062.712 | -520.445 | 35.551 | <0.001 | 0.542 | 0.527 | 108.257 |
| Annual anomalies in global land and ocean surface temperature | 1392.887 | -539.716 | -0.739 | -6.007 | <0.001 | -723.202 | -356.229 | 36.087 | <0.001 | 0.546 | 0.531 | 107.817 |
| **Climate change related variables - Air** | | | | | | | | | | | | |
| Global emission of CO_2_ | 1761.218 | -23.851 | -0.799 | -7.271 | <0.001 | -30.550 | -17.151 | 52.864 | <0.001 | 0.638 | 0.626 | 96.285 |
| Global greenhouse gas emission | 1821.144 | -18.047 | -0.812 | -7.613 | <0.001 | -22.889 | -13.206 | 57.953 | <0.001 | 0.659 | 0.648 | 93.459 |
| **Human development related variables** | | | | | | | | | | | | |
| HDI | 2833.414 | -2644.465 | -0.778 | -6.780 | <0.001 | -3441.007 | -1847.923 | 45.971 | <0.001 | 0.605 | 0.592 | 100.559 |
| Life expectancy | 4345.122 | -47.563 | -0.767 | -6.557 | <0.001 | -62.378 | -32.749 | 42.994 | <0.001 | 0.589 | 0.575 | 102.589 |
| Expected years of schooling | 2134.548 | -97.877 | -0.803 | -7.374 | <0.001 | -124.984 | -70.770 | 54.379 | <0.001 | 0.644 | 0.633 | 95.417 |
| Mean years of schooling | 2070.568 | -137.260 | -0.740 | -6.024 | <0.001 | -183.794 | -90.727 | 36.290 | <0.001 | 0.547 | 0.532 | 107.651 |
| Gross National Income per capita | 1732.323 | -0.054 | -0.835 | -8.307 | <0.001 | -0.067 | -0.040 | 69.011 | <0.001 | 0.697 | 0.687 | 88.085 |
| **Inequality related variables** | | | | | | | | | | | | |
| GII | -455.956 | 2859.329 | 0.790 | 7.050 | <0.001 | 2030.986 | 3687.671 | 49.698 | <0.001 | 0.624 | 0.611 | 98.179 |
| Maternal mortality ratio | 498.395 | 1.940 | 0.769 | 6.597 | <0.001 | 1.339 | 2.540 | 43.519 | <0.001 | 0.592 | 0.578 | 102.222 |
| Adolescent birth rate | 457.682 | 10.293 | 0.702 | 5.406 | <0.001 | 6.405 | 14.182 | 29.225 | <0.001 | 0.493 | 0.477 | 113.892 |
| Share of seats in parlimant (female) | 1521.211 | -27.348 | -0.849 | -8.808 | <0.001 | -33.690 | -21.007 | 77.578 | <0.001 | 0.721 | 0.712 | 84.505 |
| Share of seats in parlimant (male) | -1521.211 | 27.348 | 0.849 | 8.808 | <0.001 | 21.007 | 33.690 | 77.578 | <0.001 | 0.721 | 0.712 | 84.505 |
| Population with at least some secondary education (25 years and older) (female) | 1765.355 | -14.062 | -0.769 | -6.589 | <0.001 | -18.421 | -9.704 | 43.414 | <0.001 | 0.591 | 0.578 | 102.295 |
| Population with at least some secondary education (25 years and older) (male) | 1903.960 | -14.225 | -0.718 | -5.657 | <0.001 | -19.361 | -9.089 | 31.996 | <0.001 | 0.516 | 0.500 | 111.317 |
| Labor force participation rate (15 years and older) (female) | -1847.656 | 61.088 | 0.544 | 3.554 | 0.001 | 25.985 | 96.191 | 12.631 | 0.001 | 0.296 | 0.273 | 134.239 |
| Labor force participation rate (15 years and older) (male) | -4073.067 | 67.289 | 0.743 | 6.084 | <0.001 | 44.704 | 89.875 | 37.021 | <0.001 | 0.552 | 0.537 | 107.063 |
| **Globalization related variables** | | | | | | | | | | | | |
| KOF_GI | 1950.547 | -16.568 | -0.669 | -4.928 | <0.001 | -23.434 | -9.703 | 24.289 | <0.001 | 0.447 | 0.429 | 118.957 |
